# Effect of low dose denosumab on bone mineral density in postmenopausal women with osteoporosis after a transition from 60 mg dose: a prospective observational study

**DOI:** 10.1007/s12020-022-03230-5

**Published:** 2023-04-25

**Authors:** Aliya A. Khan, Hajar AbuAlrob, Iman M’Hiri, Dalal S. Ali, Karel Dandurand, Hosay Said, Hisham Alkassem, Yasser Hakami, Ismail Hweija, Salman Iqbal, Mihai Romanovschi, Shehryar Mehmood, Heather Zariffeh, Gordon Guyatt, Quazi Ibrahim, Romina Brignardello-Petersen, Hamza I. Syed

**Affiliations:** 1 McMaster University, Hamilton, Ontario Canada; 2 Bone Research and Education Centre, Oakville, Ontario Canada

**Keywords:** Postmenopausal osteoporosis, Denosumab, Bone mineral density, Observational study

## Abstract

**Introduction:**

Denosumab is an effective antiresorptive molecule and reduces the risk of fracture in postmenopausal osteoporosis. Cessation of denosumab therapy however is associated with rapid declines in bone mineral density (BMD), rises in bone remodeling, and an increased risk of fracture. We evaluated the effect of low dose denosumab (30 mg every 6 months) on the prevention of bone loss following a switch from standard dose (60 mg of denosumab every 6 months) in a prospective observational study.

**Methods:**

We recruited 114 women 50–90 years of age with postmenopausal osteoporosis at a moderate fracture risk without prior fragility fractures, who had been on denosumab 60 mg every 6 month. These women switched to low dose denosumab 30 mg every 6 months. Mean percentage change in lumbar spine (LS), femoral neck (FN), total hip (TH) and 1/3 distal radius (1/3RAD) BMD at 12 and 24 months were evaluated. Predictors for change in BMD were explored. Subgroup analysis for patients on denosumab 60 mg every 6 months for <3 years and for ≥3 years before switching to low dose denosumab 30 mg was evaluated.

**Results:**

At 12 months following a switch from 60 mg to 30 mg of denosumab every 6 months we observed an increase in LS BMD mean percentage change (+2.03%, 95% CI 1.18–2.88, *p* < 0.001). BMD was stable at the hip and radial sites. Age was found to be a predictor of the mean percentage change in LS BMD for the overall sample. At 24 months, there was a further increase in LS BMD mean percentage change (+3.44%, 95% CI 1.74–5.12, *p* < 0.001), with stable BMD at other skeletal sites. The 12 month mean BMD percentage change at the LS (*p* = 0.015), FN (*p* < 0.001), TH (*p* < 0.001), and 1/3 RAD (*p* < 0.001) were found to be predictors of the 24 month mean BMD percentage change. No clinical fractures were reported during 24 months of follow up.

**Conclusion:**

We observed stable BMD following a switch from denosumab 60 mg every 6 months to 30 mg every 6 months in this prospective observational study conducted in postmenopausal women at a moderate fracture risk

## Introduction

Postmenopausal osteoporosis is associated with significant morbidity and mortality [1, 2]. Denosumab, a potent antiresorptive agent, is a fully human IgG2 monoclonal antibody, and inhibits receptor activator of nuclear factor kappa B ligand (RANKL) [3]. Denosumab, in the standard dose of 60 mg subcutaneously every 6 months, has been demonstrated to reduce the risk of vertebral, nonvertebral, and hip fractures compared to placebo in the first 3 years of the FREEDOM study [4–7]. Denosumab treatment for up to 10 years was associated with low rates of adverse events, low fracture incidence compared with that observed during the original trial, and continued increases in BMD without plateau [8]. Denosumab is well tolerated with few adverse effects. Rare adverse effects of osteonecrosis of the jaw (ONJ) and atypical femoral fracture (AFF) have been reported with long-term use of denosumab [9–11].

Cessation of denosumab therapy may be considered in the presence of adverse effects and in women who have had significant gains in BMD with no prior history of fragility fracture and who are no longer at a high fracture risk [12, 13]. Cessation of denosumab has, however, been associated with a rebound rise in bone remodeling, reductions in BMD as well as a rapid loss of vertebral fracture protection [14, 15].

In a post hoc analysis of the data from the FREEDOM and FREEDOM extension trials multiple (>1) vertebral fractures were more commonly seen in individuals stopping denosumab in comparison to individuals stopping placebo (60.7% vs 38.7%) *p* < 0.05. The risk of multiple vertebral fractures was higher in those who had already experienced a prior vertebral fracture as well as those demonstrating rapid bone loss [15]. The rates of nonvertebral fractures were similar in the group stopping denosumab in comparison with individuals stopping placebo at 2.8, and 3.8 (per 100 participant-years), respectively [15].

Several case reports and case series have described the increased risk of fracture following cessation of denosumab [16–25] A longer duration of denosumab use prior to cessation has been associated with more significant bone loss as well as a greater number of vertebral fractures [26–28]. Both oral and intravenous bisphosphonate therapy has not been shown to fully prevent BMD declines following cessation of denosumab as published in small case reports and case series [29–31].

The efficacy of IV zoledronate in preventing bone loss following cessation of denosumab therapy has been evaluated in multiple clinical trials [32–35]. Although IV zoledronate prevents bone loss when given after discontinuation of short-term denosumab therapy, zoledronate was not able to fully prevent rises in bone turnover and bone loss when administered after long-term denosumab therapy [32, 36, 37].

Varying doses of denosumab have previously been evaluated in a 24-month randomized placebo-controlled study in 412 postmenopausal females with osteopenia or osteoporosis [36]. Denosumab was administered in varying doses (6, 14, or 30 mg every 3 months or placebo or 14, 60, 100, or 210 mg every 6 months or open-label alendronate 70 mg weekly). Denosumab increased BMD at all sites (LS, TH, FN, 1/3RAD) and decreased bone turnover markers in comparison to placebo in all doses at 24 months. There was a change in the LS BMD of greater than or equal to alendronate with all doses of denosumab except the 14 mg dose given every 6 months. At this dose, the change at the LS was less than that seen with alendronate [36].

The benefits of using a lower dose of denosumab instead of cessation of therapy include ongoing inhibition of RANKL thereby preventing the formation of excess osteoclasts (OCs) from preosteoclasts. It may also be effective in preventing the formation of osteoclasts from osteomorphs which have recently been described [38]. Bisphosphonates do not share this mechanism of action and do not inhibit RANKL and thereby would not be able to prevent the formation of OCs from osteomorphs. Low dose denosumab may also be associated with a lower risk of ONJ and AFF. A dose-dependent relationship between ONJ and denosumab has been observed in the oncology patient population in comparison to osteoporosis patients and higher doses of denosumab are associated with a higher incidence of ONJ in oncology patients [9]. It is possible that lower doses of denosumab may be associated with a lower risk of ONJ or AFF than the standard dose of 60 mg every 6 months, however this requires further study.

In this study, we evaluated BMD in patients receiving low dose denosumab 30 mg every 6 months following a switch from denosumab 60 mg every 6 months.

## Materials and methods

### Patients

Women between the ages of 50 to 90 years with postmenopausal osteoporosis were recruited from a tertiary referral center of excellence in metabolic bone disease from April 2019 to April 2020. All consecutive patients meeting the enrollment criteria and not meeting any of the exclusion criteria were considered for the study. A total of 307 patients who were on denosumab 60 mg every 6 months were screened of whom 114 were included in the study. The fracture risk was calculated using the CAROC (Canadian Association of Radiologists and Osteoporosis Canada) assessment tool [2], at the time of inclusion in the study. The CAROC risk calculator, developed in 2005, integrates gender, age, and BMD as well as the presence of a prior fragility fracture and prolonged glucocorticoid use [2]. Patients who had a moderate fracture risk and were no longer in the high risk category and had no history of a prior fragility fracture were offered the option to participate in this study. Moderate fracture risk is defined as a 10–20% risk of major osteoporotic fracture over the next 10 years. We included ambulatory women with postmenopausal osteoporosis confirmed by BMD criteria by dual-energy x-ray absorptiometry (DXA) technology with a T-score of ≤−2.5 at the LS or TH, who had previously been using denosumab 60 mg every 6 months for at least a year and who, due to side effects or concerns of potential side effects, preferred to switch to a lower dose of denosumab 30 mg every 6 months.

Inclusion criteria also included a body mass index between 18.5 and 33 kg/m^2^ as well as a normal lab profile (serum calcium corrected for albumin, parathyroid hormone, 25-hydroxyvitamin D, serum phosphorus, alkaline phosphatase (ALP), thyroid-stimulating hormone, hemoglobin, white blood cell, and platelet count). We excluded women with (1) spinal abnormalities which would have an impact on the BMD assessment from L1–L4, including abnormal vertebrae appearance, or the presence of sclerosis or scoliosis, (2) prior fragility fracture, (3) other skeletal disorders including Paget’s disease, (4) liver disease, malignancy, or a myeloproliferative disorder, or endocrine disorders that can have an impact on skeletal health, including hypercortisolism, hyperthyroidism, hyperparathyroidism, hypoparathyroidism, malabsorption, growth hormone excess or deficiency, (5) calculated estimated glomerular filtration rate of <15 mL/min, (6) previous intolerance to denosumab, (7) anticonvulsant therapy, and (8) oral or inhaled steroid use on a daily basis in the past 12 months. Following informed consent, participants who had previously been on denosumab 60 mg twice yearly and were at a moderate fracture risk were observed if they chose to switch to 30 mg twice yearly.

### Design

We conducted an observational prospective study evaluating BMD in patients receiving low dose denosumab following a switch from denosumab 60 mg every 6 months. We followed patients on low dose denosumab 30 mg every 6 months for 24 months and reported outcomes at 12 months and 24 months. The study was approved by Veritas IRB Inc. and it was conducted in accordance with the local Good Clinical Practice procedures for quality control [39].

### Procedure

Patients who had chosen to take low dose denosumab signed an informed consent and were enrolled in the study. Patients had a full clinical evaluation including medical history and physical exam. A 3-site BMD with DXA technology, conducted by International Society for Clinical Densitometry (ISCD) certified technologists, was completed at baseline, 12 months, and 24 months post-baseline. Denosumab 30 mg every 6 months was administered by research staff at the Bone Research and Education Centre (BREC). If patients were not able to return to the BREC, then the low dose denosumab could also be administered by their family physician. The 30 mg dose of denosumab was delivered by administering approximately half of the 60 mg prefilled syringe and the remainder of the dose was discarded. Calcium was obtained from dietary sources and vitamin D supplements were advised based on vitamin D levels. The target 25-hydroxyvitamin D level was 75–125 nmol/L. The patient lab profile was monitored at baseline and repeated at 12 and 24 months.

### Outcomes

The primary outcome was the percentage change in the LS BMD from baseline, at 12 and 24 months. All BMD measures were performed at the same location using the same DXA machine at baseline and follow up. A key secondary outcome was the percentage change in TH BMD at 12 months and 24 months. Other secondary outcomes included mean BMD percentage change in FN and 1/3RAD site at 12 and 24 months. Serum alkaline phosphatase was also captured at baseline and at month 12 and 24 following the switch to low dose denosumab. Safety endpoints were the overall safety and tolerability of low dose denosumab 30 mg every 6 months in women with postmenopausal osteoporosis as assessed by the presence of adverse events. These were captured on the clinical history obtained at the time of assessment. Clinical fractures were captured as an adverse event. Patients experiencing back pain or height loss of more than 1 cm had spinal X-rays completed for assessment of vertebral deformities.

#### Statistical analysis

Descriptive summaries are presented as mean ± standard deviation or as median and intervals for continuous variables, and as counts and percentage (%) for categorical data. Univariate and unadjusted data analyses were conducted for the mean percentage change in BMD for the overall sample at 12 months and 24 months. Potential predictors for the mean percentage change in BMD at the LS, FN, TH, and 1/3RAD at 12 months and 24 months were explored using linear regression analysis. The percentage change in BMD at 12 months was an additional predictor of the percentage change in BMD at 24 months. We evaluated the following predictors for the overall sample and for the subgroups: (1) the use of full dose denosumab for 3 or more years (yes or no), (2) age at the start of 30 mg denosumab (continuous), and (3) bisphosphonate use prior to the use of full dose denosumab (yes or no). We report the coefficient, 95% confidence interval (CI) and *p* value for each predictor. After identifying significant predictors (*p* < 0.05), we conducted an adjusted linear regression analysis for the percentage change in BMD at the (LS, FN, TH, and 1/3 RAD). We present the mean percentage change with 95% CI. We compared the adjusted and unadjusted models using the Akaike Information Criterion, Bayesian Information Criterion, and deviance. We present *p* value for model comparison. Statistical analysis was conducted using the statistical software analysis “R” and statistical significance was determined if *p* < 0.05.

## Results

### Patient characteristics

We enrolled 114 women with postmenopausal osteoporosis. The mean age at baseline was 68 years and the median duration of full-dose denosumab 60 mg every 6 months prior to the switch to low dose denosumab was 30 months (Table 1). Among the 114 participants, 78 patients had received denosumab 60 mg every 6 months for less than 3 years (group A) whereas 36 patients had received denosumab 60 mg every 6 months for longer than 3 years (group B) before switching to the lower dose. At baseline, 94 patients (82.5%) had been on bisphosphonates prior to enrollment (94/114). The vast majority of patients were Caucasian (95.5%) with 4.5% of patients being of Arab, Chinese or of South East Asian ancestry. Three patients (2.6%) were current smokers (3/114), 35 patients (30.7%) were past smokers, and 76 patients (66.7%) had never smoked (76/114). Patients were followed from the time of their enrollement for up to 24 months. Of the 114 women, 11 were lost to follow up at 24 months (9.6%).

**Table 1:** Patients baseline characteristics

	Total (*N* = 114)	Less than 3 years before switching (Group A) (*N* = 78)	3 years or more before switching (Group B) (*N* = 36)
Age at start of low dose denosumab (mean, SD years)	68.2 (6.5)	67.1 (6.46)	70.4 (6.18)
Duration of full dose denosumab (adjusted) (median, IQR months)	30 (18; 40.5)	20.5 (15.25; 24)	44 (37.75; 52.25)
Bisphosphonate use (percentage; *n*)	82.5% (94)	83.3% (65)	80.5% (29)
BMI (kg/m^2^) 20 or more (%; *n*)	82.5% (94)	87.2% (68)	77.8% (28)
Smoking (%; *n*)			
*Past smoker*	30.7% (35)	25.6% (20)	41.7% (15)
*Current*	2.6% (3)	2.6% (2)	2.8% (1)
*Never*	66.7% (76)	71.8% (56)	55.6% (20)
BMD LS baseline (mean, SD g/cm^2^, *n* = 113)	0.88 (0.14)	0.86 (0.12)	0.93 (0.18)
Baseline LS T-score (mean, SD)	−1.7 (1.1)	−1.8 (1.0)	−1.3 (1.2)
BMD FN baseline (mean, SD g/cm^2^, *n* = 114)	0.66 (0.09)	0.67 (0.09)	0.64 (0.08)
Baseline FN T-score (mean, SD)	−2.0 (0.5)	−1.9 (0.6)	−2.1 (0.4)
BMD TH baseline (mean, SD g/cm^2^, *n* = 114)	0.78 (0.08)	0.79 (0.09)	0.76 (0.07)
Baseline TH T-score (mean, SD)	−1.4 (0.6)	−1.4 (0.6)	−1.6 (0.5)
BMD 1/3RAD baseline (mean, SD g/cm^2^, *n* = 98)	0.62 (0.07)	0.62 (0.08)	0.61 (0.08)
Baseline 1/3RAD T-score (mean, SD)	−1.5 (1.0)	−1.5 (1.1)	−1.5 (0.7)

*BMI* body mass index, *BMD* bone mineral density, *LS* lumbar spine, *FN* femoral neck, *1/3RAD* distal third radius, *IQR* Interquartile range, *SD* standard deviation

### Change in BMD at 12 months—univariate and unadjusted analysis

At 12 months post baseline, there was an increase in the LS BMD mean percentage change (+2.03% BMD, 95% CI 1.18 to 2.88, *p* < 0.001). Changes in BMD at the FN, TH or 1/3 RAD sites in the overall sample were not statistically significant (Table 2).

**Table 2:** Mean percentage change in BMD at 12 and 24 months, overall and per group based on full dose denosumab duration

Mean percentage change in BMD at 12 months									
	Total (*N* = 114)			Less than 3 years before switching (Group A) (*N* = 78)			3 years or more before switching (Group B) (*N* = 36)		
	%	95% CI	*p* value	%	95% CI	*p* value	%	95% CI	*p* value
% Change BMD LS	2.03	1.18 to 2.88	0.00001*	1.66	0.62 to 2.71	0.002*	2.70	1.20 to 4.21	0.001*
% Change BMD FN	0.23	−0.81 to 1.26	0.664	−0.09	−1.33 to 1.14	0.880	0.85	−1.11 to 2.82	0.386
% Change BMD TH	0.59	−0.07 to 1.26	0.079	0.75	−0.07 to 1.56	0.073	0.30	−0.90 to 1.49	0.616
% Change BMD 1/3 RAD	0.08	−0.75 to 0.90	0.855	−0.18	−1.06 to 0.69	0.679	0.62	−1.24 to 2.48	0.500
Mean percentage change in BMD at 24 months									
% Change BMD LS	3.44	1.74 to 5.12	0.0002*	3.21	1.33 to 5.10	0.001*	4.49	−0.49 to 9.48	0.070
% Change BMD FN	−0.08	−1.79 to 1.63	0.929	−0.37	−2.39 to 1.65	0.710	1.34	−1.42 to 4.10	0.290
% Change BMD TH	0.03	−1.24 to 1.30	0.962	−0.05	−1.53 to 1.43	0.945	0.42	−2.31 to 3.13	0.730
% Change BMD 1/3 RAD	−0.05	−1.22 to 1.12	0.929	−0.15	−1.24 to 0.95	0.787	0.37	−4.99 to 5.72	0.873

Data are presented as (mean, 95%)

*BMD* bone mineral density, *LS* lumbar spine, *FN* femoral neck, *RAD* radius

*Statistical significance

Amongst patients who had been on the full dose denosumab (60 mg every 6 months) for less than 3 years before switching (group A), there was a rise in the LS mean percentage change BMD (+1.66% 95% CI 0.62 to 2.71, *p* = 0.0023) with no statistically significant change in BMD at the other skeletal sites (Table 2).

**Fig. 1:**
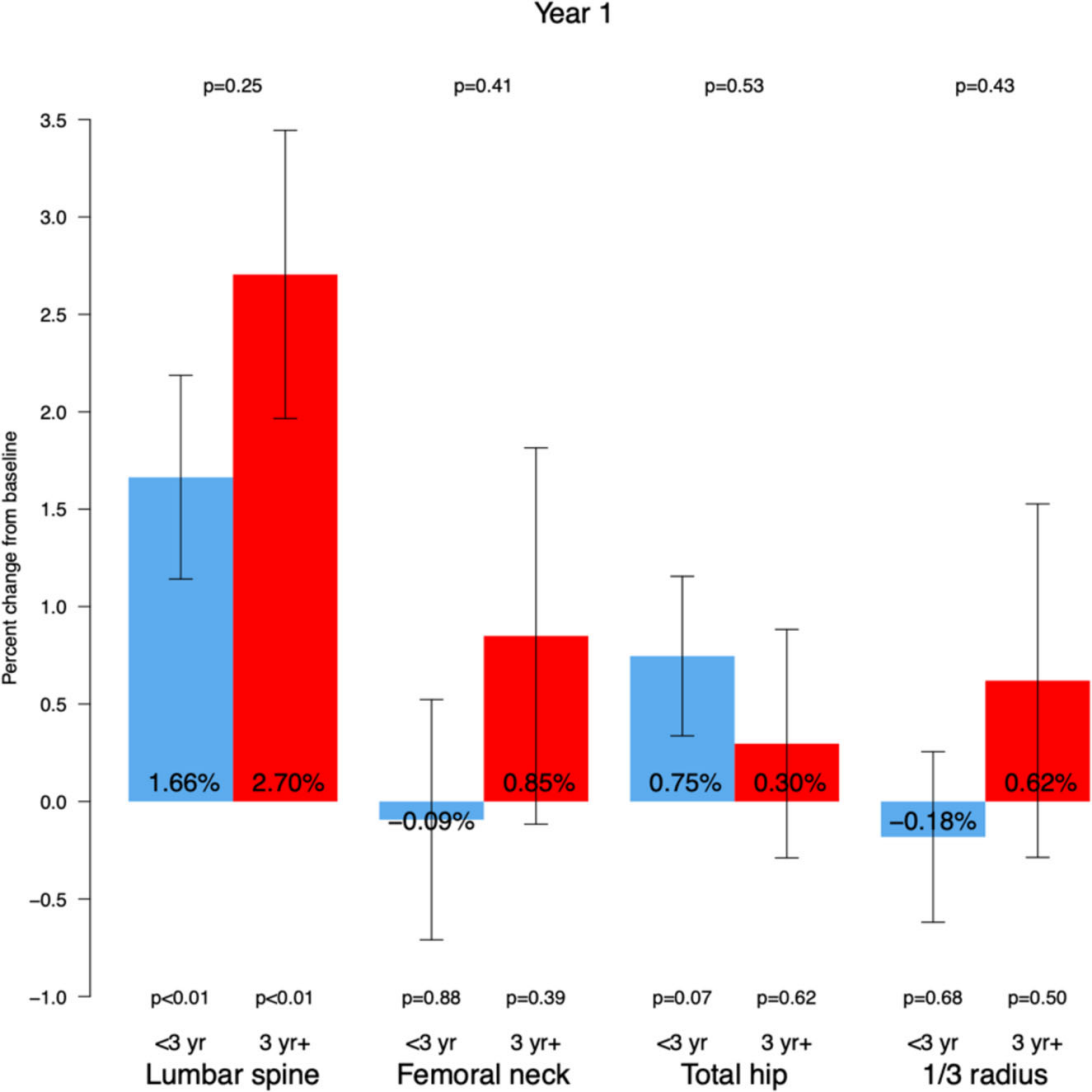
Mean % change in BMD at 12 months describes the changes in BMD values 12 months after the switch from denosumab 60 mg q 6 months to denosumab 30 mg q 6 months at the lumbar spine, femoral neck, total hip, and distal third radial sites. Blue: group A (*N* = 78), red: group B (*N* = 36)

Amongst patients who had been on full dose denosumab for ≥3 years (group B) before switching, there was a rise in BMD at the LS mean percentage change (+2.70% BMD, 95% CI 1.20–4.21, *p* < 0.001) with no change in BMD at the other sites (Table 2). We did not find a difference in the BMD response between group A and group B (Fig. 1).

### Predictors of percentage change in BMD at 12 months

#### Overall sample

We explored the following predictors for the mean percentage change in BMD at 12 months post baseline: use of full dose denosumab for 3 years or more (yes or no), age at baseline and prior bisphosphonate use (yes or no). We found age to be a predictor of the mean percentage change in LS BMD for the overall sample (*p* = 0.005) (Table 3). No other predictors for the mean percentage change in BMD in group A or group B were identified (Table 3).

**Table 3:** Unadjusted association between mean percentage change in BMD and predictors of interest at 12 months

Predictors	Coefficient	95% CI	*p* value
Outcome: LS 12 months			
*Overall sample*			
Use of full dose denosumab 3 years or more (yes or no)	1.04	0.74 to 2.82	0.25
Age at start of 30 mg denosumab (continuous)	0.13	0.001 to −0.25	0.0048
Bisphosphonate use (yes or no)	0.56	−1.64 to 2.75	0.61
*Less than 3 years before switching*			
Age at start of 30 mg denosumab (continuous)	0.15	−0.01 to 0.31	0.065
Bisphosphonate use (yes or no)	0.16	−2.61 to 2.94	0.91
*3 years or more before switching*			
Age at start of 30 mg denosumab (continuous)	0.047	−0.20 to 0.29	0.70
Bisphosphonate use (yes or no)	1.33	−2.42 to 5.09	0.47

Outcome: FN 12 months			
*Overall sample*			
Use of full dose denosumab 3 years or more (yes or no)	0.94	−1.25 to 3.13	0.39
Age at start of 30 mg denosumab (continuous)	0.15	−12.58 to −0.87	0.052
Bisphosphonate use (yes or no)	1.7	−0.95 to 4.33	0.20
*Less than 3 years before switching*			
Bisphosphonate use (yes or no)	1.035	−2.25 to 4.32	0.53
*3 years or more before switching*			
Age at start of 30 mg denosumab (continuous)	0.13	−0.18 to 0.45	0.39
Bisphosphonate use (yes or no)	2.98	−1.79 to 7.75	0.21

Outcome: TH 12 months			
*Overall sample*			
Use of full dose denosumab 3 years or more (yes or no)	−0.45	−1.86 to 0.96	0.53
Age at start of 30 mg denosumab (continuous)	0.0086	−0.09 to 0.11	0.87
Bisphosphonate use (yes or no)	0.40	−1.32 to 2.11	0.64
*Less than 3 years before switching*			
Age at start of 30 mg denosumab (continuous)	0.050	−0.08 to 0.18	0.44
Bisphosphonate use (yes or no)	0.19	−1.99 to 2.38	0.86
*3 years or more before switching*			
Age at start of 30 mg denosumab (continuous)	−0.041	−0.23 to 0.15	0.67
Bisphosphonate use (yes or no)	0.67	−2.28 to 3.63	0.64

Outcome: 1/3 RAD 12 months			
*Overall sample*			
Use of full dose denosumab 3 years or more (yes or no)	0.80	−0.97 to 2.57	0.37
Age at start of 30 mg denosumab (continuous)	0.082	−0.05 to 0.21	0.21
Bisphosphonate use (yes or no)	−0.98	−3.09 to 1.12	0.35
*Less than 3 years before switching*			
Age at start of 30 mg denosumab (continuous)	0.043	−0.09 to 0.18	0.52
Bisphosphonate use (yes or no)	−0.58	−3.00 to 1.84	0.63
*3 years or more before switching*			
Age at start of 30 mg denosumab (continuous)	0.17	−0.18 to 0.52	0.33
Bisphosphonate use (yes or no)	−1.32	−5.64 to 2.99	0.53

### Change in BMD at 24 months—univariate and unadjusted analysis

At 24 months post baseline, there was an increase in the LS BMD mean percentage change for the overall sample (+3.44% BMD, 95%CI 1.74 to 5.12, *p* < 0.001), with no change in mean BMD for the other skeletal sites in overall sample (Table 2).

In group A an increase in LS BMD mean percentage change (+3.21% BMD, 95% CI 1.33 to 5.10, *p* = 0.0014) was observed with no change at the hip or 1/3 RAD sites (Table 2).

**Fig. 2:**
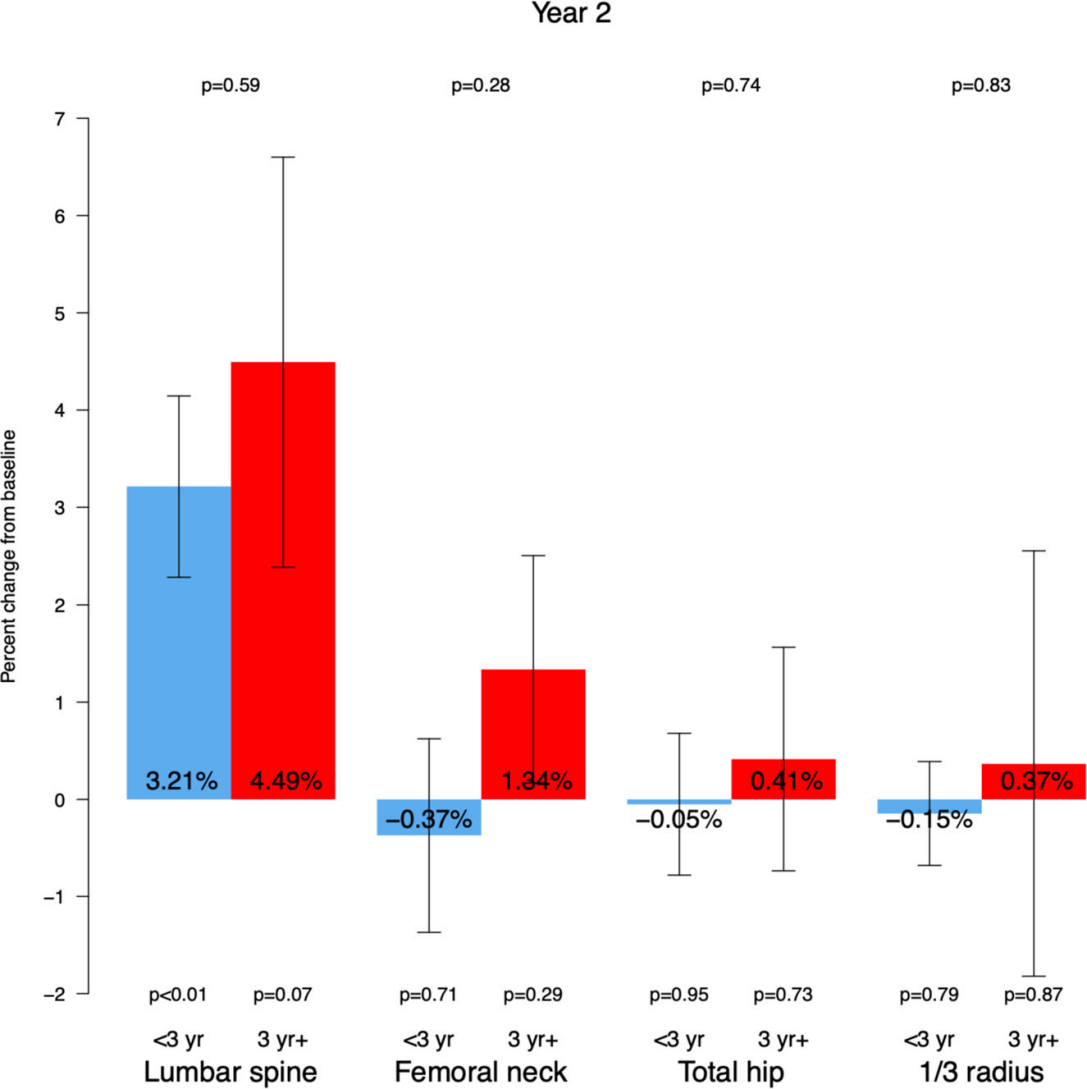
Mean % change in BMD at 24 months describes the changes in BMD values 24 months after the switch from denosumab 60 mg q 6 months to denosumab 30 mg q 6 months at the lumbar spine, femoral neck, total hip, and distal third radial sites. Blue: group A (*N* = 78), red: group B (*N* = 36)

In group B no change in mean BMD percentage change at the LS, FN, TH, or 1/3 RAD was observed (Table 2). There was no difference in percentage change in BMD at the LS, FN, TH, or 1/3 RAD between group A and group B (Fig. 2).

### Predictors of percentage change in BMD at 24 months

We explored the following predictors for the percentage change in BMD at 24 months: use of full dose denosumab for 3 years or more (yes or no), age (continuous), prior bisphosphonate use (yes or no), and 12-month percentage change in BMD. We found the 12 month mean BMD percentage change at the LS (*p* = 0.015), FN (*p* < 0.001), TH (*p* < 0.001), and 1/3 RAD (*p* < 0.001) to be a predictor of the 24 month mean BMD percentage change at the respective sites. A similar pattern was observed for patients in group A. For patients in group B, the mean percentage change at the LS BMD at 12 months was a predictor of mean percentage change in LS BMD at 24 months (*p* < 0.001), and mean percentage change in TH BMD at 12 months was a predictor (*p* = 0.013) of mean percentage change in TH BMD at 24 months. No other predictors were identified for mean 1/3RAD BMD percentage change.

### Adjusted models for percentage change in BMD at 24 months

Adjusted models for percentage change in mean BMD at 24 months for significant predictors (as identified above) was completed. The mean percentage change for LS BMD adjusted for LS BMD at 12 months was +2.13% (95% CI −0.09–4.34), FN BMD mean percentage change adjusted for FN BMD at 12 months was +0.43% (95% CI −1.21–2.08). The BMD mean percentage change adjusted for 12 months TH BMD was −0.39% (95% CI −1.54–0.77) and the 1/3RAD BMD mean percentage change adjusted for 12 months 1/3RAD BMD was 0.54% (95% CI −0.57–1.65). Adjusted models for mean LS, FN, and 1/3RAD BMD percentage change was a better fit than the unadjusted models.

### Change in ALP from baseline at 12 and 24 months

We explored the changes in ALP level in our cohort, as a marker of bone remodeling. The baseline ALP was obtained and was repeated at 12 and 24 months following transition from the standard dose (60 mg) to the low dose (30 mg) denosumab. Large variability was noted in the data, however, there was an absolute average increase in ALP in group A at 12 months which was statistically significant. There was no significant change in ALP at 12 months for group B. Similarly, there was a significant increase in ALP from baseline to 24 months in group A but not in group B. There was no statistically significant difference in average changes in ALP at 12 and 24 months compared to baseline between group A and B (Table 4).

**Table 4:** Mean Changes in ALP at baseline, 12 months, and 24 months

Changes in ALP (U/L)	Total sample (*N*)	Less than 3 years before switching (Group A) *N* (%)	3 years or more before switching (Group B) *N* (%)	*p* value for comparison between A and B
	114	78 (68.4)	36 (31.6)	
Baseline *(NR: 35–122* *U/L)*	55.1 (52.1, 58.0), 101	54.7 (52.1, 57.4), 68	55.7 (48.5, 63.0), 33	
12 months	61.0 (56.3, 65.6), 87	59.8 (56.0, 63.7), 56	63.1 (51.9, 74.2), 31	
24 months	65.3 (59.2, 71.5), 43	64.2 (58.3, 70.1), 36	71.3 (47.0, 95.5), 7	
Change from baseline at 12 months	6.0 (2.2, 9.9), 80	4.9 (2.0, 7.8), 51	8.0 (−1.4, 17.4), 29	0.542
Change from baseline at 24 months	9.6 (4.2, 15.0), 38	8.3 (3.1, 13.5), 31	15.3 (−3.5, 34.1), 7	0.508
Change from 12 months at 24 months	5.0 (−1.3, 11.2), 25	6.1 (−1.2, 13.5), 21	−1.3 (−3.7, 1.2), 4	0.074

Data are presented as mean (95% CI), *N*

### Adverse events

No adverse events were noted in this study. Fractures were captured as adverse events. There were no fractures reported during the 24 months of observation in this study evaluating postmenopausal women at a moderate fracture risk.

## Discussion

This study provides evidence that a switch from the standard 60 mg dose of denosumab to a low dose of 30 mg every 6 months may prevent bone loss and has not been associated with an increased risk of clinical fracture in postmenopausal women at a moderate fracture risk. This dosing regimen has not been evaluated previously in phase 2 clinical trials.

Cessation of denosumab therapy may be considered following long term therapy in postmenopausal women not at a high fracture risk [12]. However, it is necessary to initiate other antiresorptive treatment options if denosumab therapy is stopped in order to prevent declines in BMD and an increase in the risk of fracture.

A recent prospective cohort study evaluated the duration of denosumab exposure prior to discontinuing denosumab therapy on the change in BMD following a switch to IV zoledronate and concluded that a duration of greater than 3 years of denosumab prior use was associated with a significant decrease in LS BMD with no significant changes at the FN BMD [32, 40].

In contrast, in our study, switching from denosumab 60 mg to low dose denosumab 30 mg every 6 months was associated with maintanence of BMD at the hip and radial sites and showed small improvements in BMD at the LS regardless of the duration of previous standard dose denosumab therapy. We are conducting a follow-up study regarding the effectiveness of IV zoledronate in maintaining BMD after 24 months of low dose denosumab therapy. Low dose denosumab may serve as a bridge to transitioning to bisphosphonate therapy and this requires further evaluation.

Our subgroup analyses suggested similar changes in BMD in women who had been on standard dose denosumab for less than 3 years in comparison to women who had been on standard dose denosumab for 3 or more years. In our study BMD was maintained at all sites independent of the duration of prior treatment with standard dose denosumab 60 mg every 6 months. Furthermore, it has been described that prior bisphosphonate therapy may attenuate the rebound rise in bone remodeling observed following cessation of denosumab therapy [41]. However, in our linear regression analysis prior bisphosphonate use did not impact the effects on BMD following a reduction in the dose of denosumab at any of the skeletal sites.

Older patients in our study had a greater increase in BMD than younger patients in response to the switch from standard dose to low dose denosumab. We believe that this may be a reflection of the fact that older individuals may have had a higher rate of bone remodeling and therefore responded more robustly to low dose denosumab therapy [42].

Although vertebral fractures have been reported as early as 7 months after the last denosumab dose [43, 44], no patients with a moderate fracture risk experienced clinical fragility fractures in our study.

In our study, small rises in ALP were noted in group A. These rises in ALP were not associated with declines in BMD. A statistically significant change in ALP was not seen in group B and this may be a reflection of the smaller number of patients enrolled in group B.

Rarely, AFF and ONJ [45, 46] may occur in patients on long-term denosumab therapy and concerns about these potential side effects [47] may limit patient acceptance of long term pharmacologic intervention with standard dose denosumab. Offering a low dose option may be more acceptable to patients who have reached treatment targets with standard denosumab therapy and may be effective in preventing further declines in BMD [14].

Denosumab 60 mg every 6 months, suppresses bone turnover until the end of the dosing interval in the majority of patients [48]. As denosumab, a potent RANKL inhibitor, prevents differentiation of osteoclast precursor cells into OCs, an accumulation of osteoclast precursor cells develops during denosumab therapy, as recently described [13]. These precursor cells differentiate into OCs following cessation of denosumab therapy and this may partly explain the reversibility of the effects of denosumab. We hypothesized that low dose of denosumab 30 mg every 6 months, will result in partial suppression of RANKL and would therefore prevent the accumulation of a larger pool of precursor cells.

Recently fission of OCs into osteomorphs has been described in the animal model as occurring in the presence of RANKL inhibition [38]. Upon withdrawal of RANKL inhibition, these osteomorphs rapidly fuse to form bone-resorbing OCs. Although the process of fission and fusion was shown in an animal model it is possible that a similar process occurs in humans. Bisphosphonates may not be the ideal approach to prevent bone resorption following cessation of denosumab therapy as bisphosphonates do not inhibit RANKL activity and therefore would not prevent the formation of OCs from osteomorphs [49, 50]. Low dose denosumab may result in partial inhibition of RANKL and thus would prevent the formation of excess OCs from osteomorphs. This could explain why the low dose denosumab was effective in preventing bone loss, wheras bisphosphonates have inconsistently prevented bone loss following denosumab cessation.

### Strengths and limitations

The strengths of our study are in its prospective observational design. This was a single-center study with close follow-up. Limitations include a small sample size, a non-randomized controlled trial design as well as the absence of a control group consisting of the standard dose of denosumab 60 mg every 6 months. As reported in the literature, prior bisphosphonate use may decrease rebound osteoclastogenesis following denosumab discontinuation [41]. When exploring predictors of BMD changes at 12 months and 24 months following initiation of low dose denosumab prior bisphosphonate use was not shown to be a predictor of BMD change. However, nonbisphosphonate users comprised less than 20% of our cohort which may be a limitation in evaluating the effects of prior bisphosphonate exposure. There were no reported clinical fractures in any of the patients during the study period. Morphometric vertebral fractures were not evaluated by spine X-rays in asymptomatic patients. The study was however underpowered to detect differences in fracture risk. Another limitation of the study is the fact that the 30 mg dose of denosumab was administered by research staff or the patients’ family physician and was an estimated dose as denosumab is not marketed in a 30 mg vial. Furthermore, bone turnover markers were limited to evaluation of ALP.

Our study is ongoing, and we will also be evaluating the skeletal response to a switch from low dose denosumab to bisphosphonate therapy.

## Conclusions

This is the first study evaluating the effects of low dose denosumab on BMD in postmenopausal women following a switch from the standard dose of denosumab 60 mg every 6 months. Postmenopausal women with a moderate fracture risk receiving denosumab 30 mg every 6 months maintained BMD. Clinical fractures following a switch from denosumab 60 mg to 30 mg every 6 months were not observed.

Low dose denosumab may be a valuable option after long-term standard dose denosumab therapy, in postmenopausal osteoporosis in women who have achieved significant gains in BMD and no longer require standard dose therapy. It may also be a more acceptable option in women who have concerns regarding potential long term side effects of full-dose therapy.
